# Intensity-curvature functional based digital high pass filter of the bivariate cubic B-spline model polynomial function

**DOI:** 10.1186/s42492-019-0017-6

**Published:** 2019-08-02

**Authors:** Carlo Ciulla, Grace Agyapong

**Affiliations:** 1 Faculty of Information Systems, Visualization, Multimedia, and Animation, University of Information Science and Technology, St. Paul the Apostle, Partizanska B.B., Ohrid, 6000 Republic of North Macedonia; 2 Faculty of Communication Networks and Security, University of Information Science and Technology, St. Paul the Apostle, Partizanska B.B., Ohrid, 6000 Republic of North Macedonia

**Keywords:** Intensity-curvature functional, High pass filter, B-spline, Particle swarm optimization, K-space

## Abstract

This research addresses the design of intensity-curvature functional (ICF) based digital high pass filter (HPF). ICF is calculated from bivariate cubic B-spline model polynomial function and is called ICF-based HPF. In order to calculate ICF, the model function needs to be second order differentiable and to have non-null classic-curvature calculated at the origin (0, 0) of the pixel coordinate system. The theoretical basis of this research is called intensity-curvature concept. The concept envisions to replace signal intensity with the product between signal intensity and sum of second order partial derivatives of the model function. Extrapolation of the concept in two-dimensions (2D) makes it possible to calculate the ICF of an image. Theoretical treatise is presented to demonstrate the hypothesis that ICF is HPF signal. Empirical evidence then validates the assumption and also extends the comparison between ICF-based HPF and ten different HPFs among which is traditional HPF and particle swarm optimization (PSO) based HPF. Through comparison of image space and k-space magnitude, results indicate that HPFs behave differently. Traditional HPF filtering and ICF-based filtering are superior to PSO-based filtering. Images filtered with traditional HPF are sharper than images filtered with ICF-based filter. The contribution of this research can be summarized as follows: (1) Math description of the constraints that ICF need to obey to in order to function as HPF; (2) Math of ICF-based HPF of bivariate cubic B-spline; (3) Image space comparisons between HPFs; (4) K-space magnitude comparisons between HPFs. This research provides confirmation on the math procedure to use in order to design 2D HPF from a model bivariate polynomial function.

## Introduction

Recently, we reported a methodology to design digital HPFs from three model polynomial functions. This methodology benefits from the intensity-curvature concept and is based on the calculation of the intensity-curvature functional (ICF). The ICF replaces the image intensity value with the product of the image intensity and the sum of second-order partial derivatives of the model function fitted at each pixel. We propose the ICF of the bivariate cubic B-spline model function as a HPF to extend the earlier study [1], and to reinforce the use of the intensity-curvature concept for calculating the polynomial HPF from a model polynomial function which, in the present study, is the bivariate cubic B-spline. The originality of this study is thus to hypothesize that the ICF of an image is its HPF, and to describe a mathematical model which corroborates, both theoretically and empirically, that the ICF is indeed a HPF. From the aforementioned mathematical model, it is possible to derive the equations for the input and output functions of the filter. The novel ICF-based filter is compared with the traditional HPF and the PSO-based HPF. A comparison of the image space and the k-space magnitudes indicates that the three filters behave differently. Although the data provided in this study does not allow generalization, traditional HPF filtering and ICF-based filtering are find to be superior to PSO-based filtering. Further, the images filtered with the traditional HPF are sharper than that with the ICF-based filter. The novel contributions of this study can be summarized as follows: (1) Reasoning to elucidate the mathematical conditions that the ICF needs to obey, in order to behave as a HPF; (2) Mathematical description of ICF-based HPF of the bivariate cubic B-spline; (3) Comparison between the image spaces of the three HPFs; (4) Comparison between the k-space magnitudes of the three HPFs.

This section comprises a literature review on HPF and the ICF. The originality of the approach is then discussed in terms of the implications of the mathematical model.

### Literature on ICF

HPFs are electronic devices which allow signals with a frequency higher than a particular cutoff frequency, while removing other signals with a frequency lower than the cutoff frequency. The degree of attenuation for each frequency depends on the filter design [2]. The design of digital filter algorithms can be classified into two main categories: finite impulse response (FIR) and infinite impulse response (IIR). The FIR filter design is characterized by the product of the ideal impulse response with a window function (Butterworth, Chebyshev, Kaiser, and Hamming among others) or a gradient-based optimization method [3–5]. In contrast, the IIR filter design is characterized by a non-zero impulse response function of an infinite time duration [3]. The FIR filters can be optimally designed by a careful selection of coefficients for the frequency response, which can be written as a trigonometric function of the frequency [6, 7]. The state-of-the-art design of optimization techniques for implementing FIR digital filters includes the evolutionary and swarm optimization approaches, such as the PSO algorithm [5, 8]. ICF-based digital HPFs have been recently realized [1], where they were tested and characterized using two-dimensional images, and were observed to be belonging to the category of HPFs based on the polynomial functions [9]. The ICF is a property of the model polynomial function which is fitted to the image data on a pixel-by-pixel basis. The essence idea of ICF is to replace the image intensity with the product of the image intensity and the sum of second-order partial derivatives of the model polynomial function fitted at each pixel. Since this concept is enforced on a pixel-by-pixel basis, the ICF is an image. The original hypothesis of the above study was that the ICF is a HPF. The rationale of the present study is to revisit the aforementioned hypothesis using a new model polynomial function and to validate it through the methodological approach used earlier [1]. The visual appearance of the ICF, which is a high pass (HP) filtered signal, was first observed while investigating the ICF of the bivariate linear model function. Since then, this investigation has been extended to other model polynomial functions such as bivariate cubic and Lagrange polynomials [1]. In this study, we extend the methodological approach used for the design of a digital HPF to the bivariate cubic B-spline model function. As will be discussed in section 2, the ICF and pixel intensity are set as the output and input functions of the filter, respectively. To evaluate the novel ICF-based HPF, its k-space is compared with that of the traditional HPF and the PSO-based HPF.

### Novelty of the approach used to design the digital HPF

The main issue addressed in this study is the calculation of the digital HPF from a model polynomial function. This section presents the contribution of our approach for solving it. Customarily, HPF is mathematically defined in terms of image gradients. However, fitting a model polynomial function to an image on a pixel-by-pixel basis is not sufficient because of the prevalence of the numerical value of the image intensity over the numerical value of the gradients. Therefore, the design of the HPF is envisioned here as a resampling problem that entails: (1) modeling the image intensity with a measure that embeds both image intensity and gradients and (2) allowing numerical predominance of image intensity or gradients. To this end, the first task is to devise the measure. This measure is a ratio of two conditions, non-resampling and re-sampling, and is known as the ICF [1]. As discussed in the next section, the ICF is a valid measure because it incorporates image intensity and gradients in the same function due to which it can determine the prevalence of the gradients on the image intensity under some conditions. The academic nature of this work necessitates development of a theoretical background necessary to support the empirical evidence. Therefore, the development of a theoretical framework is the second main task of the proposed approach. The fact that ICF is a HPF [1] can be demonstrated if ICF satisfies the following two constraints: (1) the output term of the transfer function (TF) of the filter is the ICF [see eq. (12)] and (2) both the input and output functions of the filter can be designed from the mathematical expression of the ICF [see eqs. (15) and (16)]. In the next section, it is proved that the model polynomial function considered here satisfies the above constraints. Both of the aforementioned constraints are consequential to the definition of the TF of the HPF. Though the theoretical framework employed in this study was used earlier [1], the unique model polynomial function (the bivariate cubic B-spline) considered here can be used to design a novel HPF. Another novel contribution of this study is the table of the comparison between the numerical value of the gradients and the numerical value of the image intensity (Table 1).

**Table 1:** Characterization of intensity-curvature functional

Image intensity gradients	f (0, 0)	f (0, 0)	f (0, 0)	f (0, 0)
ζ (α_2_, α_3_)	<<	>>	<<	<<
γ (α_2_, α_3_)	<<	<<	>>	<<
λ (α_2_, α_3_)	<<	<<	<<	>>
Behavior of ICF	image-like	gradient-like	gradient-like	gradient-like
Image intensity gradients	f (0, 0)	f (0, 0)	f (0, 0)	f (0, 0)
ζ (α_2_, α_3_)	>>	<<	>>	>>
γ (α_2_, α_3_)	>>	>>	<<	>>
λ (α_2_, α_3_)	<<	>>	>>	>>
Behavior of ICF	image-like	gradient-like	gradient-like	gradient-like
Image intensity gradients	f (0, 0)	f (0, 0)	f (0, 0)	f (0, 0)
ζ (α_2_, α_3_)	! < < AND ! >>	! > > AND ! < <	! < < AND ! >>	! < < AND ! >>
γ (α_2_, α_3_)	! < < AND ! >>	! < < AND ! >>	! > > AND ! < <	! < < AND ! >>
λ (α_2_, α_3_)	! < <	! < < AND ! >>	! < < AND ! >>	! >>
Behavior of ICF	gradient-like or image-like	gradient-like or image-like	gradient-like or image-like	gradient-like or image-like

This table can be read by considering: 1. The term in the first column (which is ζ (α_2,_ α_3_) or γ (α_2,_ α_3_) or λ (α_2,_ α_3_)); 2. The inequality sign (in the cell of the table); 3. The value of the pixel intensity f(0, 0). For instance: ζ (α_2_, α_3_) < < f(0, 0). The symbols: ‘!’, ‘<<’, ‘>>’, ‘AND’; mean different, largely smaller than, largely bigger than, and the logical and, respectively. Thus, the symbol ‘! < < AND ! >>’ reads: not largely less than and at the same time not largely greater than. Moreover, the symbol ‘! > > AND ! < <’ reads: not largely greater than and at the same time not largely smaller than

## Theory

### ICF of bivariate cubic B-spline model function

Let us consider the bivariate cubic B-spline model function as defined in eq. (1). This function benefits from 1. *The property of second-order differentiability in its domain of definition (the pixel)* and 2. *The non-null classic-curvature of the model function calculated at the origin (0, 0) of the pixel coordinate system* [10]. It uses the pixel intensity value f(0, 0) and eight neighboring pixel intensity values: f (1/2, 1/2), f (− 1/2, − 1/2), f (2/3, 2/3), f (− 2/3, − 2/3), f (− 1, − 1), f (1, 1), f (3/2, 3/2) and f (− 3/2, − 3/2).

1 $$ {\mathrm{h}}_4\left(\mathrm{x},\mathrm{y}\right)=\mathrm{f}\left(0,0\right)+{\upalpha}_3\cdot \left[\left(\frac{1}{2}\right){\left(\mathrm{x}+\mathrm{y}\right)}^3-{\left(\mathrm{x}+\mathrm{y}\right)}^2+\left(\frac{2}{3}\right)\right]+{\upalpha}_2\cdot \left[-\left(1/6\right){\left(\mathrm{x}+\mathrm{y}\right)}^3+{\left(\mathrm{x}+\mathrm{y}\right)}^2-2\left(\mathrm{x}+\mathrm{y}\right)+\left(\frac{4}{3}\right)\right] $$

where:

2 $$ {\upalpha}_2=\left[\mathrm{f}\left(0,0\right)-\mathrm{f}\left(\frac{1}{2},\frac{1}{2}\right)\right] $$

3 $$ {\upalpha}_3=\left[\mathrm{f}\left(0,0\right)-\mathrm{f}\left(-\frac{1}{2},-\frac{1}{2}\right)\right] $$

The first order partial derivatives of h_4_(x) with respect to the x and y variables are given in eq. (4):

4 $$ \left(\partial \frac{\left({h}_4\left(x,y\right)\right)}{\partial x}\right)=\left(\partial \frac{\left({h}_4\left(x,y\right)\right)}{\partial y}\right)={\alpha}_3\cdot \left[\left(\frac{3}{2}\right){\left(x+y\right)}^2-2\left(x+y\right)\right]+{\alpha}_2\cdot \left[-\left(\frac{3}{6}\right){\left(x+y\right)}^2+2\left(x+y\right)-2\right] $$

The second order partial derivatives of h_4_(x) are given in eq. (5):

5 $$ \left({\partial}^2\frac{\left({h}_4\left(x,y\right)\right)}{\partial {x}^2}\right)=\left({\partial}^2\frac{\left({h}_4\left(x,y\right)\right)}{\partial {y}^2}\right)=\left({\partial}^2\frac{\left({h}_4\left(x,y\right)\right)}{\partial x\partial y}\right)=\left({\partial}^2\frac{\left({h}_4\left(x,y\right)\right)}{\partial y\partial x}\right)={\alpha}_3\cdot \left[3\left(x+y\right)-2\right]+{\alpha}_2\cdot \left[-\left(x+y\right)+2\right] $$

Let the intensity-curvature term before interpolation be defined as:

6 $$ E\mathrm{o}=E\mathrm{o}\left(x,y\right)=\int \int f\left(0,0\right)\cdot {\left\{\left({\partial}^2\frac{\left({h}_4\left(x,y\right)\right)}{\partial {x}^2}\right)+\left({\partial}^2\frac{\left({h}_4\left(x,y\right)\right)}{\partial x\partial y}\right)+\left({\partial}^2\frac{\left({h}_4\left(x,y\right)\right)}{\partial y\partial x}\right)+\left({\partial}^2\frac{\left({h}_4\left(x,y\right)\right)}{\partial {y}^2}\right)\right\}}_{\left(0,0\right)} dxdy $$

From eq. (5), it follows that:

7 $$ {\left({\partial}^2\frac{\left({h}_4\left(x,y\right)\right)}{\partial {x}^2}\right)}_{\left(0,0\right)}={\left({\partial}^2\frac{\left({h}_4\left(x,y\right)\right)}{\partial {y}^2}\right)}_{\left(0,0\right)}={\left({\partial}^2\frac{\left({h}_4\left(x,y\right)\right)}{\partial x\partial y}\right)}_{\left(0,0\right)}={\left({\partial}^2\frac{\left({h}_4\left(x,y\right)\right)}{\partial y\partial x}\right)}_{\left(0,0\right)}=-2{\alpha}_3+2{\alpha}_2 $$

Therefore:

8 $$ E\mathrm{o}\left(x,y\right)=\int \int f\left(0,0\right)\left\{-2{\alpha}_3+2{\alpha}_2\right\} dxdy=4 xy\cdot f\left(0,0\right)\left\{-2{\alpha}_3+2{\alpha}_2\right\} $$

Let the intensity-curvature term after interpolation be defined as:

9 $$ {E}_{IN}={E}_{IN}\left(x,y\right)=\int \int {\mathrm{h}}_4\left(\mathrm{x},\mathrm{y}\right){\left\{\left({\partial}^2\frac{\left({h}_4\left(x,y\right)\right)}{\partial {x}^2}\right)+\left({\partial}^2\frac{\left({h}_4\left(x,y\right)\right)}{\partial x\partial y}\right)+\left({\partial}^2\frac{\left({h}_4\left(x,y\right)\right)}{\partial y\partial x}\right)+\left({\partial}^2\frac{\left({h}_4\left(x,y\right)\right)}{\partial {y}^2}\right)\right\}}_{\left(x,y\right)} dxdy $$

From eq. (5) it follows that:

10 $$ {E}_{IN}\left(x,y\right)=\int \int 4\cdot {\mathrm{h}}_4\left(\mathrm{x},\mathrm{y}\right){\left\{{\upalpha}_3\cdot \left[3\left(\mathrm{x}+\mathrm{y}\right)-2\right]+{\upalpha}_2\cdot \left[-\left(\mathrm{x}+\mathrm{y}\right)+2\right]\right\}}_{\left(x,y\right)}\mathrm{dxdy}=4\cdot \left\{f\left(0,0\right){\alpha}_3\cdot \left[3\left({x}^2y/2+x{y}^2/2\right)-2 xy\right]+f\left(0,0\right){\alpha}_2\cdot \left[-\left({x}^2y/2+x{y}^2/2\right)+2 xy\right]\right\}+4\cdot \left\{\left[\frac{3}{2}{\alpha_3}^2-\frac{1}{2}{\alpha}_3{\alpha}_2-\frac{3}{6}{\alpha}_3{\alpha}_2+\frac{1}{6}{\alpha_2}^2\right]\cdot \left[{x}^5y/5+\frac{3}{8}{x}^4{y}^2+{x}^3{y}^3/3+{x}^2{y}^4/8+{x}^4{y}^2/8+{x}^3{y}^3/3+\frac{3}{8}{x}^2{y}^4+x{y}^5/5\right]+\left[-4{\alpha_3}^2+2{\alpha}_3{\alpha}_2+\frac{4}{3}{\alpha}_3{\alpha}_2-\frac{4}{3}{\alpha_2}^2\right]\left[{x}^4y/4+{x}^3{y}^2/2+{x}^2{y}^3/2+x{y}^4/4\right]+\left[2{\alpha_3}^2-2{\alpha}_3{\alpha}_2-8{\alpha}_3{\alpha}_2+4{\alpha_2}^2\right]\left[{x}^3y/3+{x}^2{y}^2/2+x{y}^3/3\right]+\left[2{\alpha_3}^2-\frac{2}{3}{\alpha}_3{\alpha}_2+8{\alpha}_3{\alpha}_2-\frac{16}{3}{\alpha}_{2^2}\right]\left[{x}^2y/2+x{y}^2/2\right]+\left[-\frac{4}{3}{\alpha_3}^2+\frac{4}{3}{\alpha}_3{\alpha}_2-\frac{8}{3}{\alpha}_3{\alpha}_2+\frac{8}{3}{\alpha_2}^2\right]\left[ xy\right]\right\} $$

The ICF of h_4_(x, y) is defined as:

11 $$ \Delta  E\left(x,y\right)=\frac{E_0\left(x,y\right)}{E_{IN}\left(x,y\right)} $$

### Input function x[n] and output function y[n] of ICF-based HPF

The TF(x, y) of the ICF-based HPF calculated from the bivariate cubic B-spline is given in eq. (12):

12 $$ TF\left(x,y\right)=\left(\frac{Y\left(x,y\right)}{X\left(x,y\right)}\right)=\left(\frac{\Delta E\left(x,y\right)}{f\left(0,0\right)}\right) $$

It implies that:

13 $$ Y\left(x,y\right)\cdot f\left(0,0\right)=\Delta E\left(x,y\right)\cdot X\left(x,y\right) $$

14 $$ y\left[n\right]=\frac{f\left(0,0\right)\cdot {e}_{12}}{\left[4\cdot \left(f\left(0,0\right)\cdot {\alpha}_3\cdot {e}_1+f\left(0,0\right)\cdot {\alpha}_2\cdot {e}_2\right)+\omega \right]} $$

Therefore the input function x[n] and the output function y[n] of the ICF-based HPF are:

15 $$ x\left[n\right]=\frac{\omega \cdot y\left[n\right]}{\left[{e}_{12}-4y\left[n\right]\cdot \left({\alpha}_3{e}_1+{\alpha}_2{e}_2\right)\right]} $$

16 $$ y\left[n\right]=\frac{x\left[n\right]\cdot {e}_{12}}{\left[4\cdot \left(x\left[n\right]\cdot {\alpha}_3\cdot {e}_1+x\left[n\right]\cdot {\alpha}_2\cdot {e}_2\right)+\operatorname{}\omega \right)\Big]} $$

**Fig. 1:**
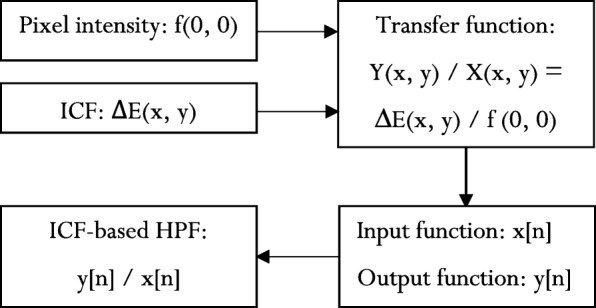
Flowchart for the calculation of ICF-based HPF

The procedure outlined in eqs. (12) through (16) is described in Fig. 1, where:

17 $$ {e}_1=\left[3\cdot \left(\frac{x^2y}{2}+\frac{x{y}^2}{2}\right)-2 xy\right] $$

18 $$ {e}_2=\left[-\left(\frac{x^2y}{2}+\frac{x{y}^2}{2}\right)+2 xy\right] $$

19 $$ {e}_{12}=4\cdot \left(-2{\alpha}_3+2{\alpha}_2\right) $$

20 $$ \omega =4\cdot \left({e}_3+{e}_4+{e}_5+{e}_6+{e}_7+{e}_8+{e}_9+{e}_{10}+{e}_{11}\right) $$

21 $$ {e}_3=\left[\frac{3}{2}{\alpha_3}^2-\frac{1}{2}{\alpha}_3{\alpha}_2-\frac{3}{6}{\alpha}_3{\alpha}_2+\frac{1}{6}{\alpha_3}^2\right] $$

22 $$ {e}_4=\left[\frac{x^5y}{5}\operatorname{}+\frac{3}{8}{x}^4{y}^2+\frac{x^3{y}^3}{3}+\frac{x^2{y}^4}{8}+\frac{{\mathrm{x}}^4{y}^2}{8}+\frac{x^3{y}^3}{3}+\frac{3}{8}{x}^2{y}^4\operatorname{}+\frac{x{y}^5}{5}\right] $$

23 $$ {e}_5=\left[-4{\alpha_3}^2+2{\alpha}_3\ {\alpha}_2+\frac{4}{3}\ {\alpha}_3\ {\alpha}_2-\frac{4}{3}{\alpha_2}^2\ \right] $$

24 $$ {e}_6=\left[\frac{x^4y}{4}+\frac{x^3{y}^2}{2}+\frac{x^2{y}^3}{2}-\frac{x{y}^4}{4}\right] $$

25 $$ {e}_7=\left[2{\alpha_3}^2-2{\alpha}_3\ {\alpha}_2-8{\alpha}_3\ {\alpha}_2+4{\alpha_2}^2\right] $$

26 $$ {e}_8=\left[\frac{x^3y}{3}+\frac{x^2{y}^2}{2}+\frac{x{y}^3}{3}\right] $$

27 $$ {e}_9=\left[2\ {\alpha_3}^2-\left(\frac{2}{3}\right)\ {\alpha}_3\ {\alpha}_2+8\ {\alpha}_3\ {\alpha}_2-\left(\frac{16}{3}\right){\alpha_2}^2\right] $$

28 $$ {e}_{10}=\left[\frac{\ {x}^2y}{2}+\frac{x{y}^2}{2}\right] $$

29 $$ {e}_{11}=\left[-\frac{4}{3}{\alpha_3}^2+\frac{4}{3}{\alpha}_3{\alpha}_2-\frac{8}{3}{\alpha}_3{\alpha}_2+\frac{8}{3}{\alpha_2}^2\right]\cdot \left[ xy\right] $$

### Characterization of ICF of bivariate cubic B-spline model polynomial function (H42D)

**Fig. 2:**
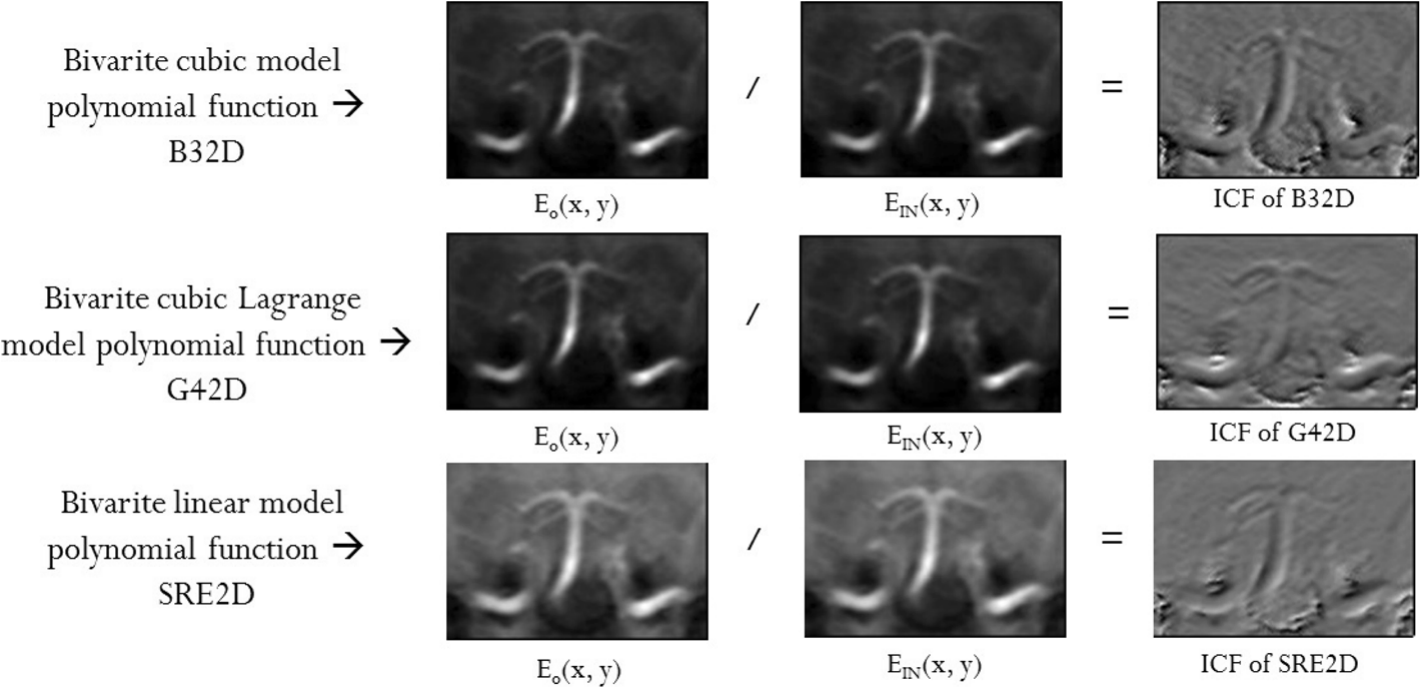
Intensity-curvature functional of the three model functions. The intensity-curvature functional (ICF) is the ratio of the intensity curvature terms (ICT) before (E_o_ (x, y)) and after interpolation (E_IN_ (x, y)). Here, the ICF appears as the HPF even though the ICTs are not HPFs. These correspond to the cases when the convolutions of pixel intensity values in the defining equation of the ICF are not responsible for the creation of the high pass (HP) filtering effect, which shows up in the ICF images. The HPF-like appearance of the ICF in such cases can be attributed to the effect of the ratio between ICTs. This figure demonstrates that the gradients embedded in the formulation of the ICTs are not the reason for this appearance

The behavior of the ICF is highly dependent on the predominance of gradients on the pixel intensity values. Thus, an immediate comparison between the ICF and the image gradients along the X and Y directions indicates that the similarities between the ICF and the HPF may be attributed to the finite differences (gradients) that appear in the formulae of ICF and HPF [11]. However, further investigations show that there exists a case where the two intensity curvature terms (ICTs) appear similar to the departing image (Fig. 2). This happens when the image intensity f(0, 0) prevails (both in the numerator and denominator) over the gradient-like components in the ICF formula.

In order to characterize the ICF as a HPF, mathematical formalism is needed so to categorize the ICF as the output function of the equation that defines the transfer function TF of the filter. This mathematical formalism needs to break the ICF formula in components and to analyze each component separately. The mathematical form of the ICF of the bivariate cubic B-spline model polynomial function (H42D) is:

30 $$ \Delta E\left(x,y\right)=4 xy\cdot f\left(0,0\right)\left\{-2{\alpha}_3+2{\alpha}_2\right\}/\Big\{4\cdot \left\{f\left(0,0\right){\alpha}_3\cdot \left[3\left({x}^2y/2+x{y}^2/2\right)-2 xy\right]+f\left(0,0\right){\alpha}_2\cdot \left[-\left({x}^2y/2+x{y}^2/2\right)+2 xy\right]\right\}+4\cdot \left\{\left[\frac{3}{2}{\alpha_3}^2-\frac{1}{2}{\alpha}_3{\alpha}_2-\frac{3}{6}{\alpha}_3{\alpha}_2+\frac{1}{6}{\alpha_2}^2\right]\left[{x}^5y/5+\frac{3}{8}{x}^4{y}^2+{x}^3{y}^3/3+{x}^2{y}^4/8+{x}^4{y}^2/8+{x}^3{y}^3/3+\frac{3}{8}{x}^2{y}^4+x{y}^5/5\right]+\left[-4{\alpha_3}^2+2{\alpha}_3{\alpha}_2+\frac{4}{3}{\alpha}_3{\alpha}_2-\frac{4}{3}{\alpha_2}^2\right]\left[{x}^4y/4+{x}^3{y}^2/2+{x}^2{y}^3/2+x{y}^4/4\right]+\left[2{\alpha_3}^2-2{\alpha}_3{\alpha}_2-8{\alpha}_3{\alpha}_2+4{\alpha_2}^2\right]\left[{x}^3y/3+{x}^2{y}^2/2+x{y}^3/3\right]+\left[2{\alpha_3}^2-\frac{2}{3}{\alpha}_3{\alpha}_2+8{\alpha}_3{\alpha}_2-\frac{16}{3}{\alpha_2}^2\right]\left[{x}^2y/2+x{y}^2/2\right]+\left[-\frac{4}{3}{\alpha_3}^2+\frac{4}{3}{\alpha}_3{\alpha}_2-\frac{8}{3}{\alpha}_3{\alpha}_2+\frac{8}{3}{\alpha_2}^2\right]\left[ xy\right]\right\}=\frac{\mathrm{f}\left(0,0\right)\cdot {e}_{12}}{\left\{4\cdot \left[\left(\mathrm{f}\left(0,0\right)\cdot {\alpha}_3\cdot {e}_1+\mathrm{f}\left(0,0\right)\cdot {\alpha}_2\cdot {e}_2\right)\right]+\operatorname{}\omega \right)\Big\}}=\frac{\mathrm{f}\left(0,0\right)\cdot \upzeta \left({\alpha}_2,{\alpha}_3\right)}{\left[\mathrm{f}\left(0,0\right)\cdot \upgamma \left({\alpha}_2,{\alpha}_3\right)+\uplambda \left({\alpha}_2,{\alpha}_3\right)\right]} $$

In general, as can be deduced from the ratio of eqs. (8) and (10), the ICF can be reduced to the following representation:

31 $$ \Delta E\left(x,y\right)=\frac{\mathrm{f}\left(0,0\right)\cdot \upzeta \left({\alpha}_2,{\alpha}_3\right)}{\left[\mathrm{f}\left(0,0\right)\cdot \upgamma \left({\alpha}_2,{\alpha}_3\right)+\uplambda \left({\alpha}_2,{\alpha}_3\right)\right]}=\frac{\upzeta \left({\alpha}_2,{\alpha}_3\right)}{\left\{\upgamma \left({\alpha}_2,{\alpha}_3\right)+\left[\frac{\uplambda \left({\alpha}_2,{\alpha}_3\right)}{\mathrm{f}\left(0,0\right)}\right]\right\}} $$

where it is posited that:

32 $$ \upzeta \left({\alpha}_2,{\alpha}_3\right)={e}_{12} $$

33 $$ \upgamma \left({\alpha}_2,{\alpha}_3\right)=\left[4\cdot {\alpha}_3\cdot {e}_1+4\cdot {\alpha}_2\cdot {e}_2\right] $$

34 $$ \uplambda\ \left({\alpha}_2,{\alpha}_3\right)=\omega $$

The gradient-like appearance of the ICF is thus determined by the numerical prevalence of the terms that are function of the convolutions (ζ (α_2_, α_3_), γ (α_2_, α_3_) and λ (α_2_, α_3_)). Otherwise, when f(0, 0) prevails, the ICF appears similar to a magnetic resonance image (MRI).

In eq. (31), we assume that ζ (α_2_, α_3_) < < f(0, 0) and γ (α_2_, α_3_) < < f(0, 0) and λ (α_2_, α_3_) < < f(0, 0). In this case, the prevalent term in the numerator and denominator is the image intensity f(0, 0) (this case is illustrated in Fig. 2). On the contrary, if we assume that ζ (α_2_, α_3_) > > f(0, 0) and γ (α_2_, α_3_) > > f(0, 0) and λ (α_2_, α_3_) > > f(0, 0), the prevalent terms are ζ (α_2_, α_3_) and γ (α_2_, α_3_) and the ICF image appears as a HPF. Table 1 indicates that all the remaining cases can be reduced to either of the two listed above.

To summarize, when f(0, 0) is the prevailing term, the ICF reflects the predominant influence of the pixel intensity value f(0, 0). On the contrary, when α_2_ and α_3_ are both larger than f(0, 0), the ICF behaves as a HPF because of the gradient-like components α_2_ and α_3_.

In essence, the characterization of the ICF as a HPF acknowledges that when the convolutions of pixel intensity values in the defining equation of the ICF are tuned as the finite differences operating as gradients, they (the convolutions) create the HP filtering effect. This happens under the condition that the convolutions are prevalent on the image intensity value. To break the ICF formula into components (as illustrated in eq. (31)) and to build the table, may be extended to other model polynomial functions in order to investigate the effect of gradient-like components and image intensity on the appearance of the ICF image.

### Ethics approval and consent

The MRI scans were conducted with the sole purpose of this study and not for clinical purpose. The study participants were anonymized. Informed consent to participate in the study was obtained from the participants, and this consent allows to publish the MRI images while protecting the privacy of the participants.

## Results

### Comparison between ICF and traditional HP filtering and PSO filtering approaches

**Fig. 3:**
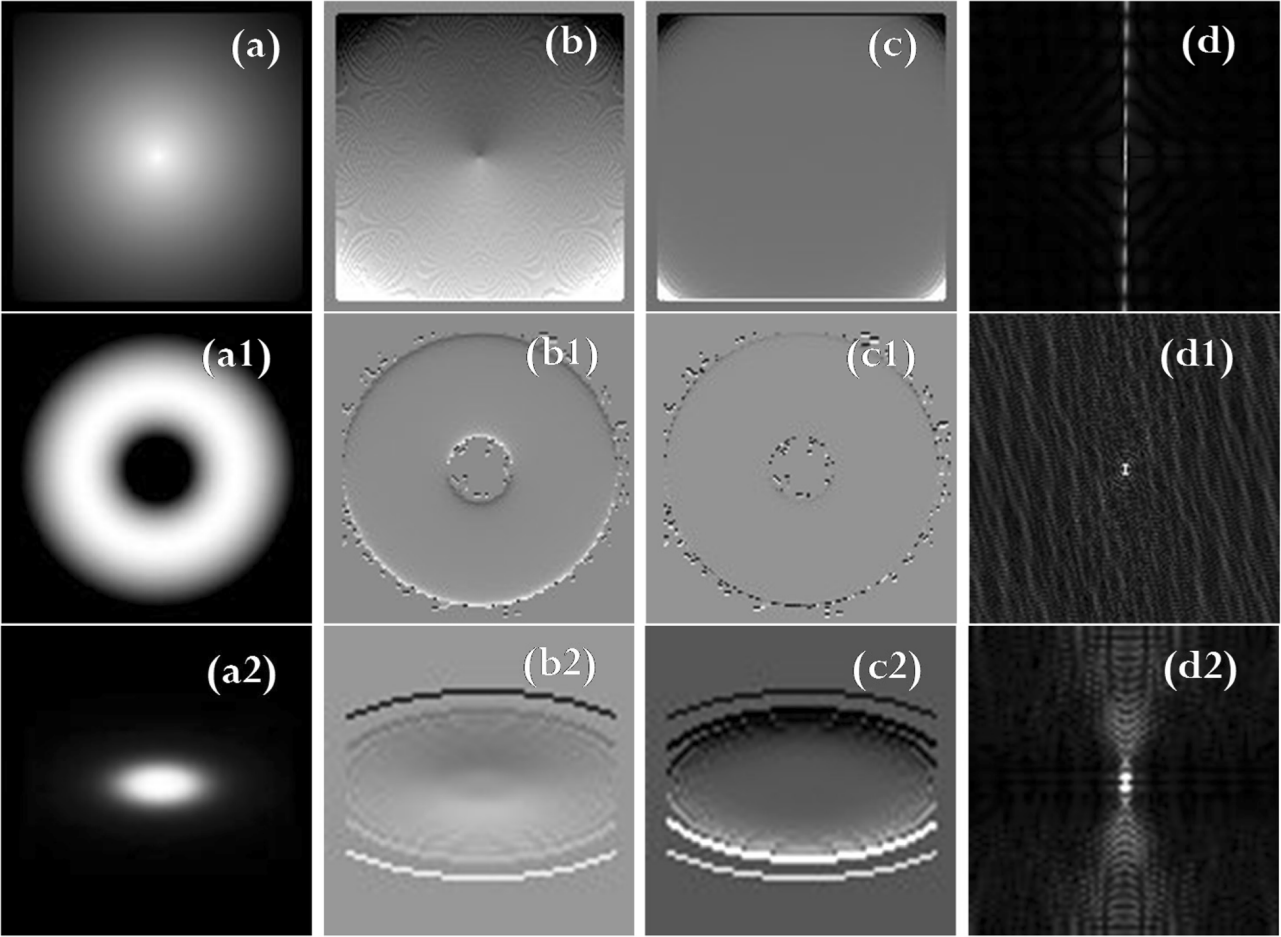
(**a**), (a1), (a2) Original images. Here, (**a**) is calculated using spherical light source, (a1) is the calculated light torus, and (a2) is the calculated elliptical light. (**b**), (b1), (b2) ICF of the bivariate cubic B-spline model function (eq. (11)). (**c**), (c1), (c2) TF of ICF-based HPF (eq. (12)). (**d**), (d1), (d2) k-space magnitude of ICF

**Fig. 4:**
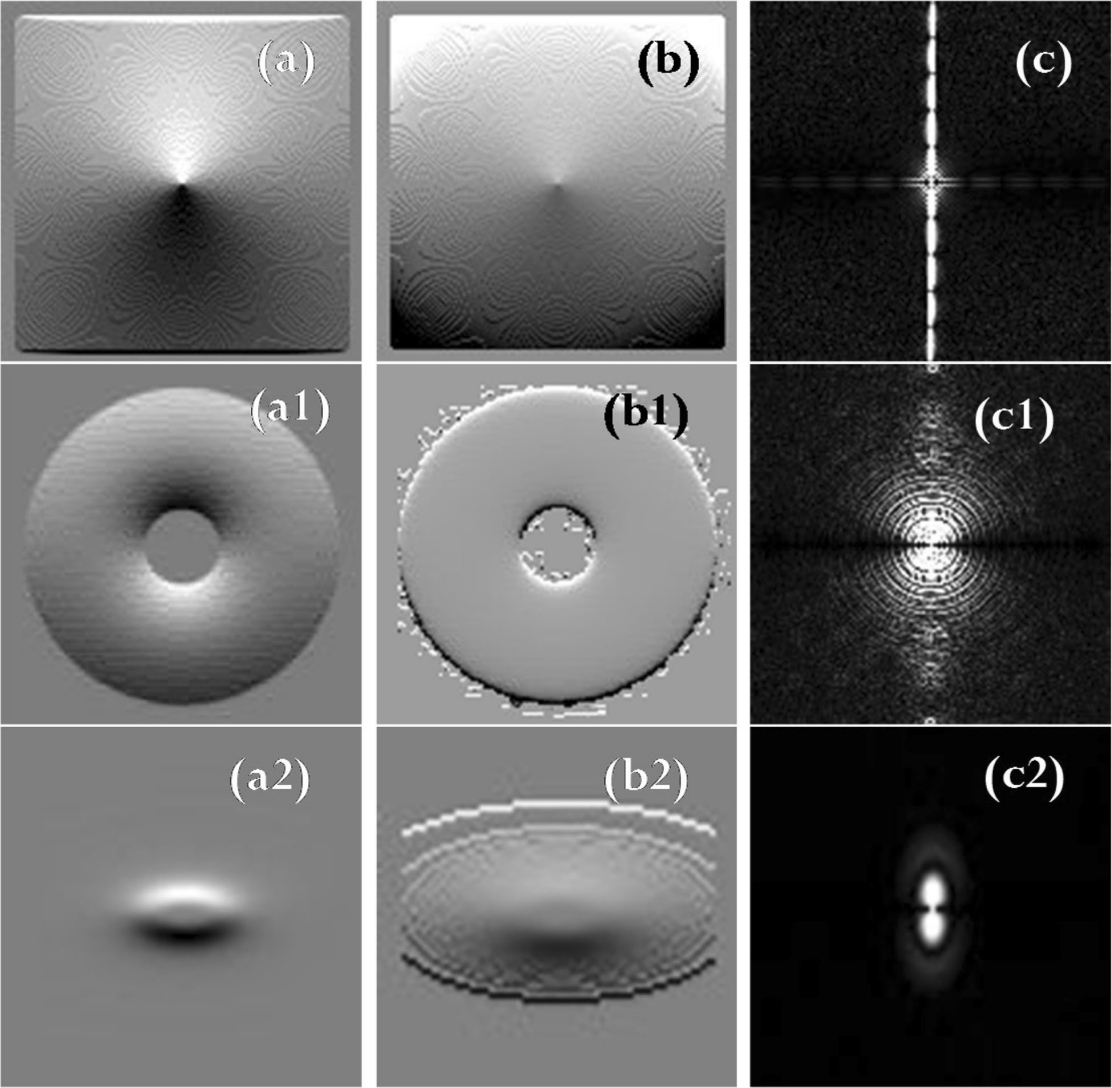
(**a**), (a1), (a2): Traditional HPF images of Figs. 3 (**a**), (a1), (a2), respectively. (**b**), (b1), (b2): Transfer function images of the traditional HPF images. (**c**), (c1), (c2): k-space magnitude of the traditional HPF images

**Fig. 5:**
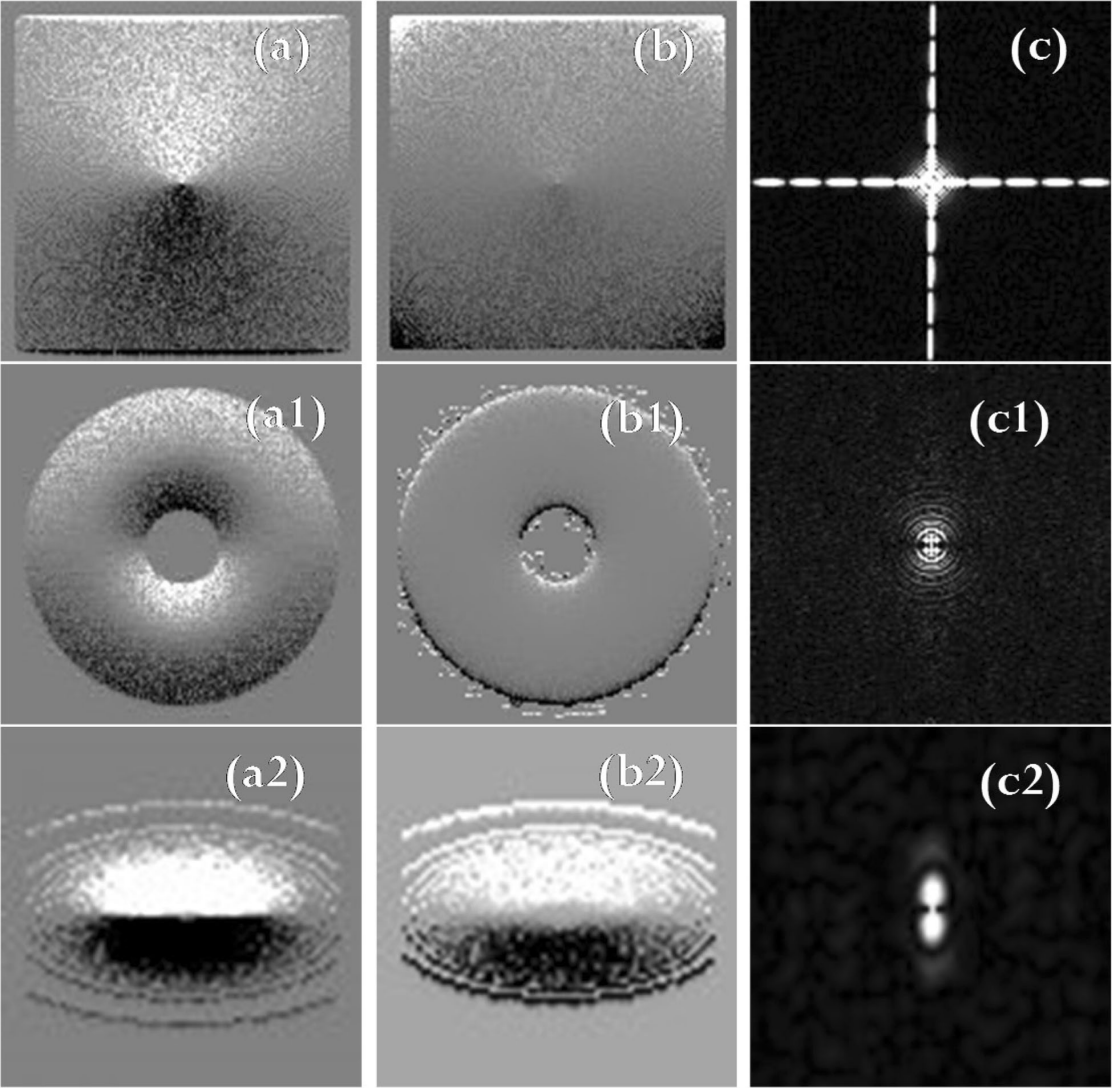
(**a**), (a1), (a2): PSO-based HP filtered images of Figs. 4(**a**), (a1), (a2), respectively. (**b**), (b1), (b2): Transfer functions of PSO filter. (**c**), (c1), (c2): k-space magnitude of the PSO based filtered images shown in (**a**), (a1), (a2), respectively

The data set consisted of ten theoretical images and two MRIs. This section illustrates, for the selected cases, the results of filtering with the ICF, the traditional HPF, and the PSO. For the remaining cases, the three filters performed in a similar manner. For each of the three filters, the TF and the k-space magnitude of the filtered images is shown in Figs. 3, 4 and 5, respectively. Moreover, to reinforce the difference between the three filters, we show the k-space magnitude images of the filtered images, the difference calculated between the filtered images, and the difference calculated between the k-space images.

It is noteworthy from Figs. 3 and 4 that the ICF images are well behaved, however, the HPF images are sharper than the ICF images. The difference between the HPF and ICF images is of particular significance, and it is remarkably visible in the k-space magnitude [compare Figs. 3(d), (d1), (d2) with Figs. 4(c), (c1), (c2), respectively].

The images presented in Figs. 3, 4, and 5 show the superiority of the traditional HPF and the ICF-based filter over the PSO-based filter, and this is noticeable if we compare the filtered images in Fig. 4(a), (a1), and (a2) with that in Figs. 5(a), (a1), and (a2), respectively. There exist similarities between the k-space magnitude of the HPF filtered images and that of the PSO filtered images (compare Fig. 4(c), (c1), and (c2) with Fig. 5(c), (c1), and 5(c2), respectively).

**Fig. 6:**
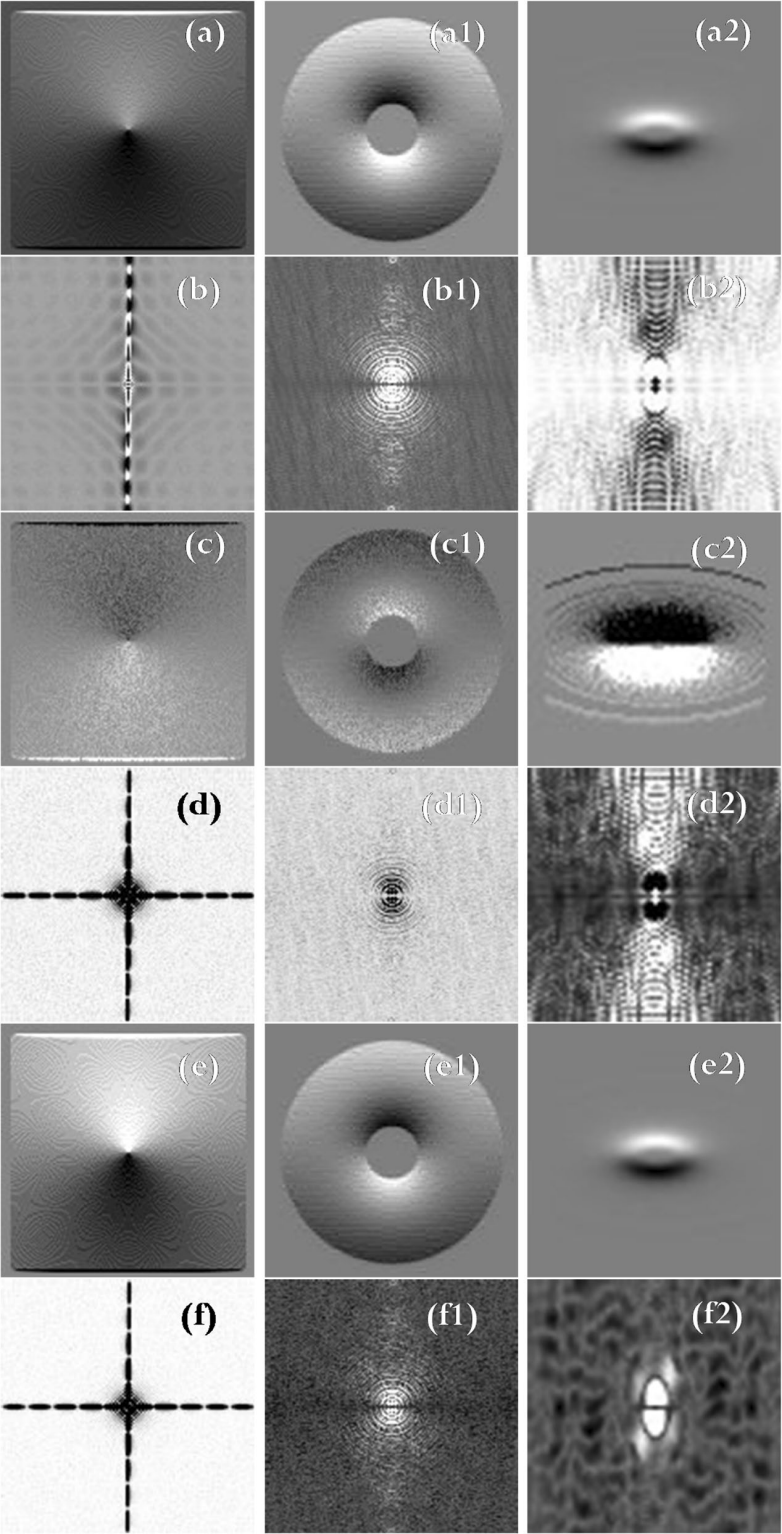
(**a**), (a1), (a2): Difference between the ICF images (Fig. 3) and the traditional HPF images (Fig. 4). (**b**), (b1), (b2): Difference between the k-space magnitude images of ICF (Fig. 3) and that of HPF (Fig. 4). (**c**), (c1), (c2): Difference between the image space of ICF (Fig. 3) and that of PSO (Fig. 5). (**d**), (d1), (d2): Difference between the k-space magnitude of ICF (Fig. 3) and that of PSO (Fig. 5). (**e**), (e1), (e2): Difference between the image space of HPF (Fig. 4) and that of PSO (Fig. 5). (**f**), (f1), (f2): Difference between the k-space magnitude of HPF image (Fig. 4) and that of PSO image (Fig. 5)

The images displayed in Fig. 6 are a clear and straightforward presentation of the existing differences between the filters discussed in this paper. These images show the difference between the image space and k-space.

### Comparison of proposed approach with similar existing methods

**Fig. 7:**
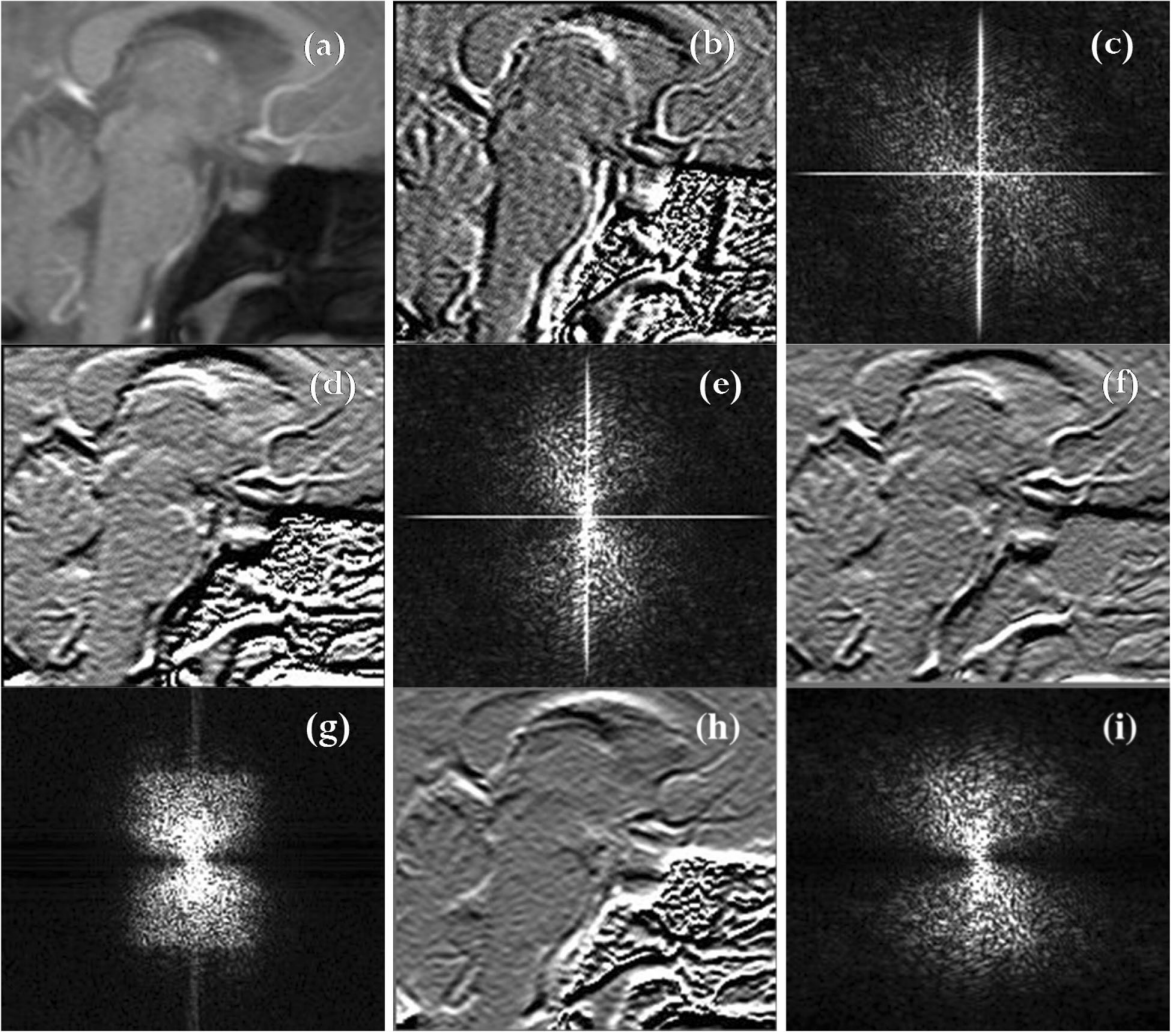
Comparison across ICF-based HPFs and traditional HPFs. (**a**) MRI. (**b**) MRI filtered with bivariate cubic polynomial model function B32D ICF-based HPF. (**c**) The k-space magnitude of (**b**). (**d**) MRI filtered with the bivariate cubic Lagrange polynomial model function G42D ICF-based HPF. (**e**) The k-space magnitude of (**d**). (**f**) MRI filtered with traditional HPF. (**g**) The k-space of (**f**). (**h**) MRI filtered with the bivariate cubic B-spline H42D ICF-based HPF. (**i**) The k-space magnitude of (**h**)

Figure 7 shows a comparison across ICF-based HPFs and the traditional HPF. The model functions and the characterization of the B32D ICF-based HPF and the G42D ICF-based HPF are reported elsewhere [1]. Through the comparison of the k-space magnitude of the HP filtered images, Fig. 7 clearly shows that these filters are not similar, and their difference can be attributed to the mathematical form of the departing polynomial from which the ICF is obtained.

**Fig. 8:**
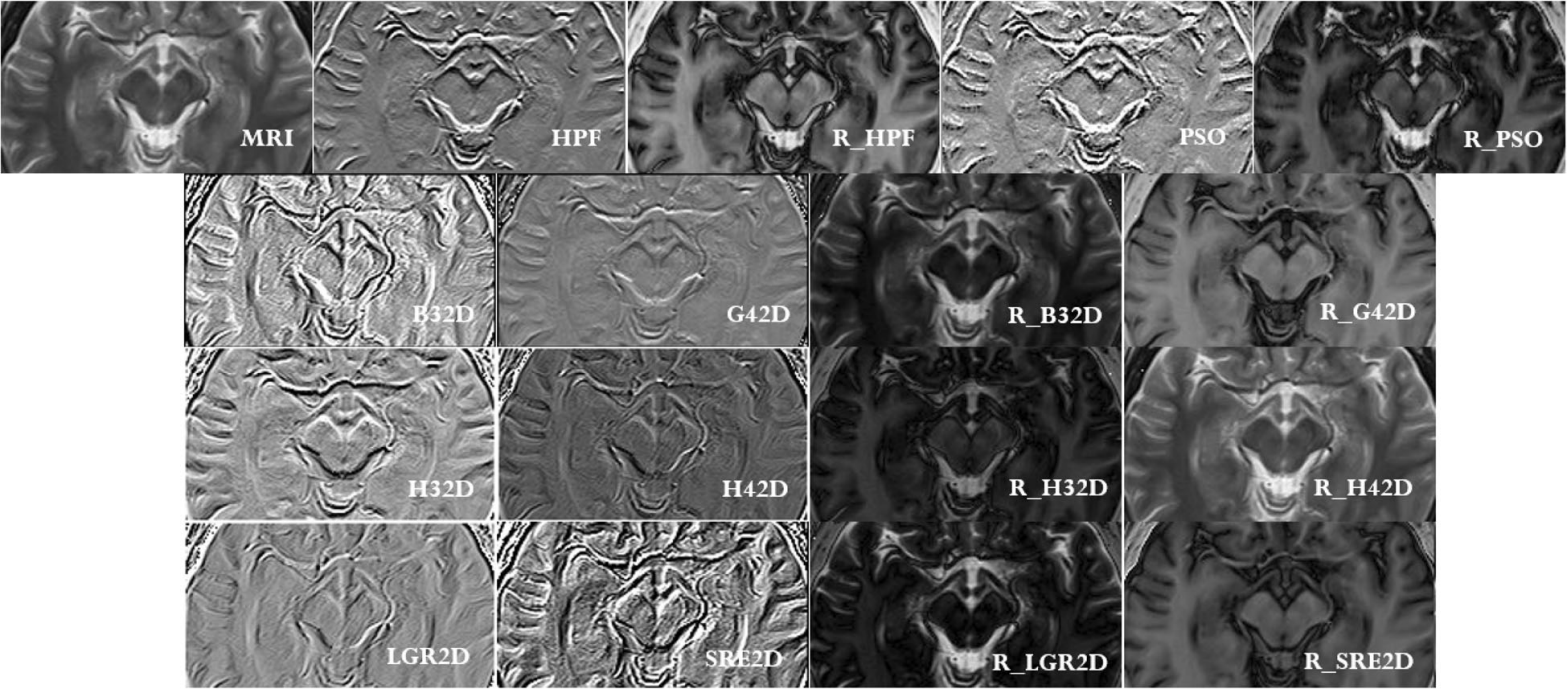
Comparative study across filtering techniques. Top row (from left to right): MRI, HPF, reconstructed MRI through HPF (R_HPF), PSO-based HPF, and reconstructed MRI through PSO (R_PSO). Second row: ICF of B32D, ICF of G42D, reconstructed MRI through ICF of B32D (R_B32D), and reconstructed MRI through ICF of G42D (R_G42D). Third row from the top: ICF of H32D, ICF of H42D, reconstructed MRI through ICF of H32D (R_H32D), and reconstructed MRI through ICF of H42D (R_H42D). Bottom row: ICF of LGR2D, ICF of SRE2D, reconstructed MRI through ICF of LGR2D (R_LGR2D), and reconstructed MRI through ICF of SRE2D (R_SRE2D)

**Fig. 9:**
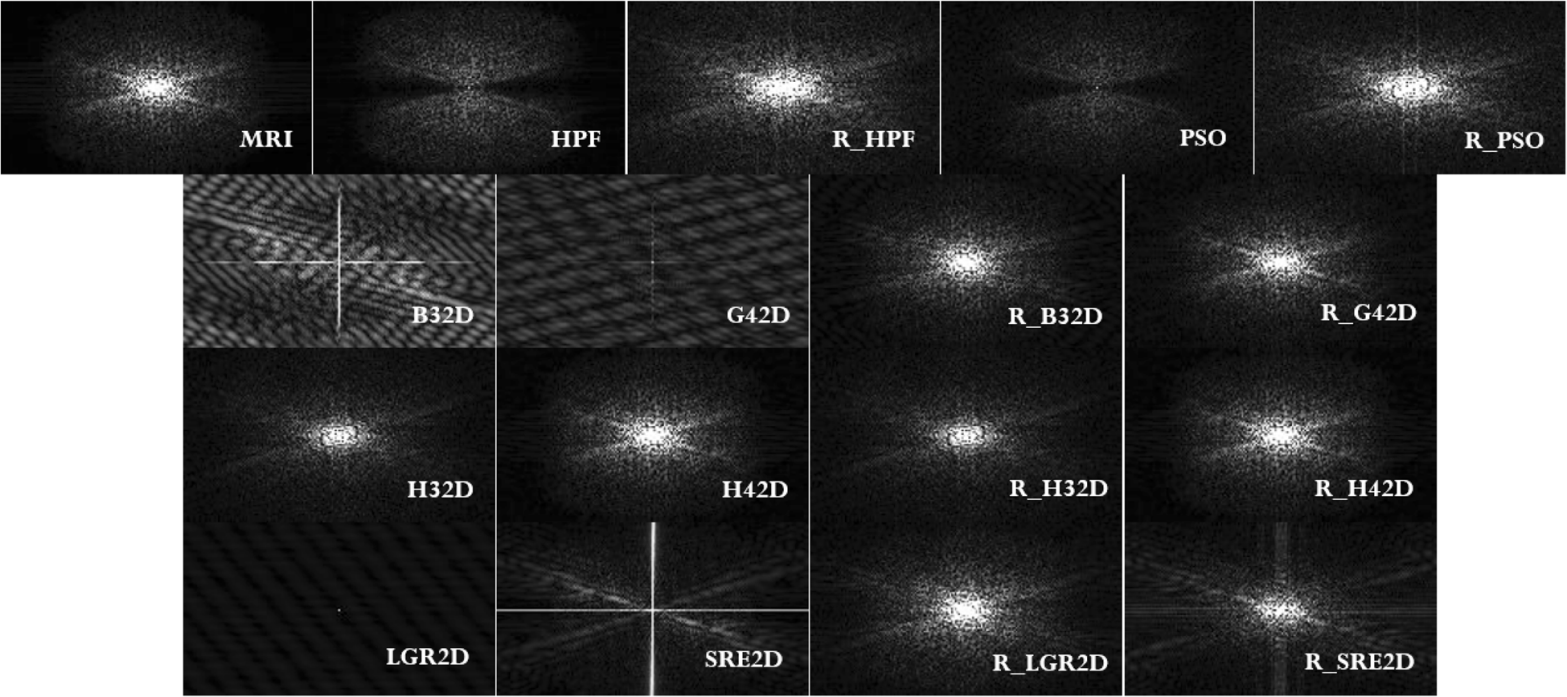
Comparative study across filtering techniques. The picture shows the respective k-space magnitude of the images displayed in Fig. 8

**Fig. 10:**
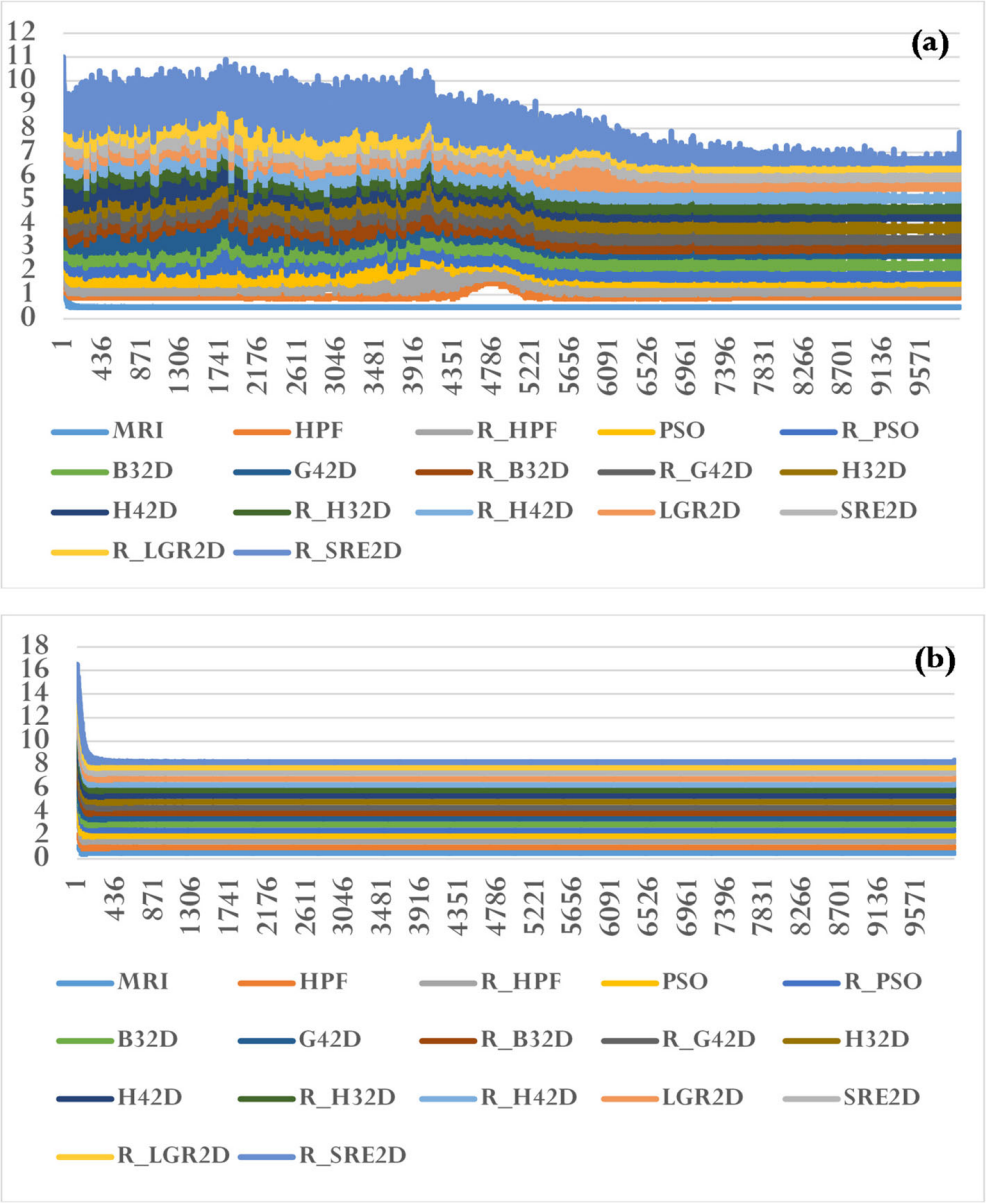
Normal distribution values, calculated using the formula $\mathrm{f}\left(\mathrm{x},\upmu, \sigma \right)=\left(\frac{1}{\sqrt{2\uppi \sigma }}\right){e}^{-\left[{\left(\mathrm{x}-\upmu \right)}^2/\left(2{\upsigma}^2\right)\right]}$, for the filtered images presented in Fig. 8 [see lines in (**a**)] and for the k-space images presented in Fig. 9 [see lines in (**b**)]. The plots show cumulative lines. Each line corresponds to an image, and displays the value of f(x, μ, σ) calculated using the value of ‘x’ (which is the histogram data of the image)

Figures 7, 8, and 9 show a comparative study between imaging modalities involving the ICF of several model functions as k-space filters. The MRIs are compared with ICFs, and the ICFs are used as k-space filters through the signal processing technique, known as the inverse Fourier transformation [12]. Figure 8 provides two important aspects of this study. The first aspect is the reproducibility of the HPF behavior of the ICF for six model polynomial functions (including the bivariate cubic B-spline which is the model used in this paper). The other aspect is to verify that the inverse Fourier transformation technique facilitates MRI signal reconstruction. The signal reconstruction highlights the vessels of the human brain. Figure 8 also presents the comparison of ICFs with traditional HPF and PSO-based HPF. Figure 9 shows the difference between the k-space of each imaging modality. Through the histogram plots of the k-space images, Fig. 10 provides a quantitative outlook of the difference between the filtering techniques.

Figures 8 and 9 show the filtered signals and their k-space magnitudes, respectively. Figure 10 shows the results of statistical analysis performed on the filtered images [see (a)] and the k-space magnitude images [see (b)]. The histogram was calculated for each image, and the corresponding value of the mean and standard deviation were also computed. The plots in Fig. 10 show cumulative lines. Each line represents an image and connects the value f(x, μ, σ) of the normal distribution obtained from each of the ‘x’ histogram values. For instance, the histogram value ‘x’ provides the value f(x, μ, σ), which is the integral of the normal distribution from -∞ to ‘x’, expressed as $\mathrm{f}\left(\mathrm{x},\upmu, \sigma \right)=\left(\frac{1}{\sqrt{2\uppi \sigma }}\right){e}^{-\left[{\left(\mathrm{x}-\upmu \right)}^2/\left(2{\upsigma}^2\right)\right]}$. The graphs shown in Figs. 10(a) and (b) are thus cumulative normal distribution values. The plots of f(x, μ, σ) confirm that the filtered signals (Fig. 8) are Gaussian distributed, whereas the k-space signals (Fig. 9) are not.

**Fig. 11:**
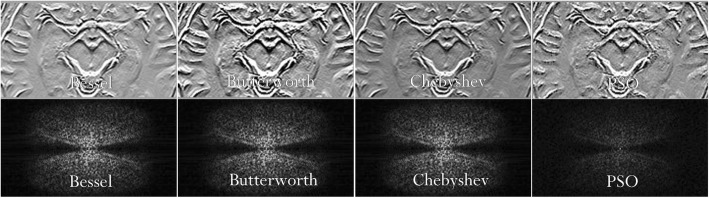
Comparison between classic HP filtering techniques (Bessel, Butterworth and Chebyshev) and the PSO-based HPF. The images on the bottom row are the k-space magnitude images of the signals located in the top row

Figure 11 shows the comparison between HP filtered signals with the aim of validating PSO-based filtering technique. Indeed, the visual appearance of the PSO-based filtered image is quite similar to the classic filtered signals obtained with Bessel, Butterworth and Chebyshev HPFs. This paper offers a comparison between PSO filtering and ten different HPF methods: traditional HPF, B32D, G42D, H32D, H42D (bivariate cubic B-Spline), LGR2D, SRE2D, Bessel, Butterworth, and Chebyshev HPFs.

## Discussion

### Applications of digital filters literature

The literature on digital filters is extensive. This section provides a compendium of studies corresponding to two most important themes. One theme is related to the effort of the scientific community towards the design of digital filters, with an emphasis on the hardware implementation. The other theme reports on the application of digital filters in academic research and industry settings. Table 2 provides a list of authors who have contributed to this topic. Study of electroencephalography (EEG) traces was motivated by the identification of interictal high frequency oscillations (HFOs). This research raised a methodological issue which comes into play when HFOs are filtered because they embed sharp transient frequency components, which result in the so-called confounding false ripples of EEG signal [13] while being filtered. Thus, the identification and quantification of false ripples was pivotal for the correct analysis of broadband activity of various frequency bands. It is also important to determine a strategy that can be used to reduce the influence of the false ripples on the analysis of the EEG signals. Therefore, this study demonstrated that the false ripple occurs during band-pass filtering of the EEG signal, and the false ripple consists of a signal whose frequency is close to the impulse response of the filter, and which appears as a transient signal similar to a short duration oscillation [13]. Another report on application of digital filters in human technology [14] demonstrated the benefits of filtering out the raw surface empirical mode decomposition (sEMD) signal while estimating the muscle forces in the human body. This study found a novel use of the HPFs that can remove up to 90% - 99% of the sEMD signal power, and this can improve the force estimates of the specific muscles in the body. Another technological application of high-pass electrical mobility filter (HP-EMF) [15], which is related to the size of nanoparticles in the gaseous media, was also reported. The HP-EMFs have shown a potential to serve as a viable technology for diverse applications ranging from the synthesis of nanomaterials to monitoring atmospheric nanoparticles. An industrial application of HPFs, i.e., a methodology for the production of metamaterials, was reported in ref. [16]. The metamaterial explored in this study consisted of engineered subwavelength microstructures, and it exhibited ‘plasmonic’ response to the electromagnetic waves in the terahertz (THz) range. This property caused the metamaterial to behave as a HPF. The purpose of the study was to demonstrate the efficiency of the THz plasmonic HPF which consists of high-aspect-ratio micron-sized wire arrays that are fabricated by micro-stereo-lithography. This study provided a method to tune the plasma frequency by adjusting the geometric parameters of the metamaterial [16]. In the context of infrared focal-plane array (IRFPA) technology, the significance of the research reported in ref. [17] was to explore the analysis of temporal HPFs. A new non-uniformity correction method called bilateral filter based temporal high-pass filter (BFTH) was proposed. The main properties of the HPF developed in this study were convergence speed and ghosting artifacts. Through comparison between algorithmic performances, the study demonstrated that the BFTH has the fastest speed of convergence and the most stable error. A study aimed towards designing and implementing computationally efficient two-dimensional FIR digital filters was reported in ref. [18]. The design procedure was based on frequency domain analysis because the Fourier domain favors the analysis of the interpolated impulse response. This study demonstrated that, as compared to an equivalent conventional FIR filter, interpolated finite impulse response (IFIR) filters require only 1/L_th_ of the adders and multipliers, while offering 1/L_th_ noise level and 1/√L_th_ coefficient sensitivity. The IFIR filter comprised of two components (called FIR sections): one generated the sparse set of impulse response values, and the other executed interpolation. The eigenfilter approach was discussed in ref. [19] as a new method for designing linear-phase FIR filters. This method minimized the quadratic cost function of the error (defined in the passband and stopband of the filter) by calculating the difference between the actual amplitude response and the desired response of the filter. It was possible to obtain the equation defining a linear system from the defining equation of the cost function. The eigenvectors, which are the parameters of the filter, could then be calculated from the definite matrix defining the linear system.

**Table 2:** Compilation of studies which explored different applications of HPFs

Authors	High-pass filters	Authors	Filters	Authors	Finite impulse response filters
Bénar et al. (2010)	Pitfalls of high-pass filtering for detecting epileptic oscillations: A technical note on ‘false’ ripples	Jing (1987)	A New Method for Digital All-Pass Filter Design	Neuvo et al. (1984)	Interpolated finite impulse response filters
				Chandra et al. (2016)	Design of hardware efficient FIR filter: A review of the state-of-the-art approaches
Potvin et al. (2003)	Less is more: high pass filtering, to remove up to 99% of the surface EMG signal power, improves EMG-based biceps brachii muscle force estimates	Saramaki (1985)	On the Design of Digital Filters as a Sum of Two All-Pass Filters		
				Vaidyanathan et al. (1987)	Eigenfilters: A new approach to least-squares FIR filter design and applications including Nyquist filters
				Pun et al. (2002)	On the design and implementation of FIR and IIR Digital Filters with variable frequency characteristics
Surawski et al. (2017)	A tunable high-pass filter for simple and inexpensive size-segregation of sub-10-nm nanoparticles				
				Sharma et al. (2016)	Performance of swarm based on optimization techniques for designing digital FIR filter: comparative study.
Wu et al. (2003)	Terahertz plasmonic high pass filter				
				Zhao et al. (1988)	A simple design of FIR filters with powers-of-two coefficients
Zuo et al. (2011)	New temporal high-pass filter nonuniformity correction based on bilateral filter				

A recent review paper [7] presented an overview of methodological approaches for designing hardware efficient finite duration impulse response (FIR) filters. A design method for FIR digital filters, which has gained considerable attention recently, is the multiplier-less filter. This filter is hardware efficient, and is designed with the optimization (minimization) of the multiplier-less operations. It was reported that the hardware efficiency of the multiplier-less filters could be achieved on the basis of various indices which indicate the number of hardware components, such as power-of-two-terms, number of adders, number of multiplier and structural adders, number of flip-flops, and zero-valued filter coefficients [7]. The theoretical and the implementation aspects of FIR and IIR variable digital filters (VDFs) was reported in ref. [20]. These filters exhibited frequency characteristics which could be controlled continuously by tuning some parameters such as the poles and the zeros of the digital filter. Two main highlights of this work were as follows: (1) Computational simplicity of the filter design which only required the calculation of the singular value decomposition of a Hankel matrix. (2) Through the aforementioned calculation, the IIR based VDF was guaranteed to be stable and the frequency response was conserved. The algorithm reported in ref. [21] focused on the design of FIR filters with powers-of-two coefficients (the 2PFIR filter). The algorithm comprised of two methods. The first method maintained the existing relationships between the coefficients of the conventional FIR and the 2PFIR filters. This method was called proportional relation-preserve (PRP) method. The second method consisted of an application of the simple symmetric-sharpening (SSS) approach. The SSS method used the PRP method as sub-filter and preserved the transition width. Further, it improved the approximation error of the PRP method. Using the PRP and SSS methods, the feasibility of designing a 2PFIR filter of length (N) greater than 200 was demonstrated.

The research work reported in ref. [5] focused on a linear phase infinite-impulse response filter, which was designed through the nature inspired population-based global optimization algorithm known as Cuckoo Search (CS) [22]. The main aspect of the study was that the controlling parameter, which quantitatively determined the ripple (and thus the amount of energy) in the desired frequency bands (both stop and pass), was experimentally set in such a way that the best performance of the filter could be obtained. The current relevance of the study can be attributed to the fact that it presented a comparative evaluation of three nature inspired optimization methods for FIR filter design, which are (1) the Cuckoo search (CS) [22], (2) the PSO [23], and (3) the artificial bee colony (ABC) [24].

### Recent literature

Recent efforts in designing a digital FIR filter can be classified into two groups. The first one utilizes L_1_-norm based optimization techniques for designing the filter, and the second uses evolutionary approaches which mimic the organizational behavior of living beings, such as the well-known PSO algorithm. The essence of the L_1_-norm based digital filter design is the formulation of the cost function, the determination of a suitable algorithm for its minimization, and the acquisition of the filter equation coefficients [25]. A recent research [26] uses genetic algorithms to solve the optimization problem of the L_1_-norm based filter design, and compares the filter with a wide array of competing techniques such as the PSO [3, 4, 8] and conventional methods such as the least-squares approach [27], the Kaiser window method [28], and the Parks McClellan algorithm [29].

One of the most compelling and state-of-the-art techniques in current digital filter design is the PSO approach. This method is essentially an optimization algorithm, and therefore it suffers from the drawback of obtaining the most suitable cost function, the most suitable parameters to optimize, and the most suitable optimization rule. As with every adaptive search, the performance of this optimization algorithm is also affected by the initially chosen parameter values, and can be stuck in a local minimum of the cost function (in such a case, the optimization does not progress). Various solutions have been proposed in the literature to promote PSO approach as the state-of-the-art technique [30–33], and these have inspired our implementation of the PSO as a simple, quick, and effective approach. Our approach depends on: (1) a least square function as the cost function of the algorithm, (2) the delta rule as the optimization algorithm and (3) cardinal parameters that were chosen on the basis of experience (particle’s position, particle’s best position, swarm’s best known position and particle’s velocity, as explained in Wikipedia).

**Fig. 12:**
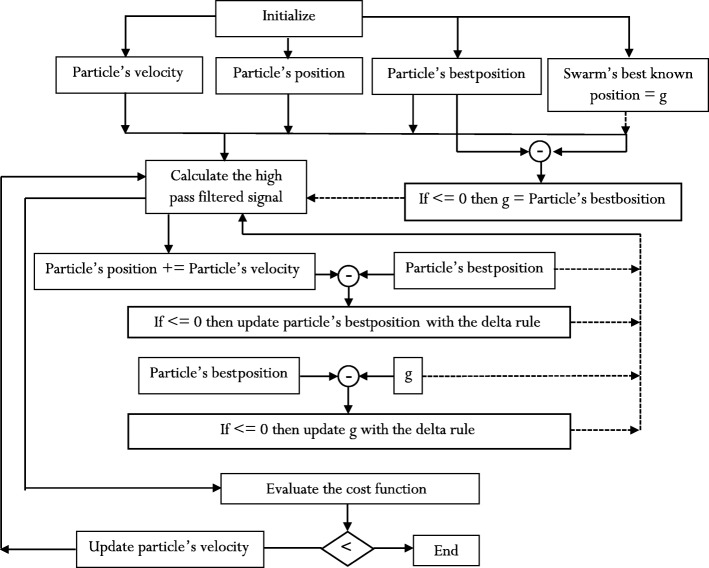
A flowchart for the implementation of particle swarm optimization

**Fig. 13:**
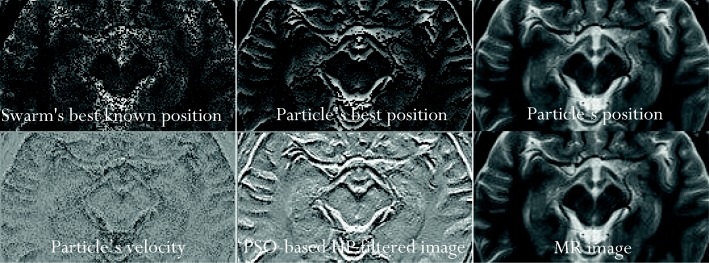
Results of the optimization process of the particle swarm optimization algorithm for one case study. The relationships between the images are presented in the flowchart in Fig. 12

In our implementation of the PSO approach, the HPF equation is developed to mimic the calculation of the particle’s velocity, which is consistent with the approach used in ref. [34]. At each iteration of the optimization algorithm, the particle’s position is updated with the sum of its current value and the value of the particle’s velocity. The initialization of the PSO optimization algorithm provides the starting values of the cardinal parameters mentioned earlier [35]. The initialization heuristics set the starting values as random numbers, whose sum is the value of the image intensity. One limitation of our implementation is premature convergence [36]. We have chosen to implement the algorithm reported in Wikipedia due to its inter-application robustness [37]. This means that the algorithm is suitable to be re-engineered with little modification so that it can be applied for optimization problems other than the calculation of HP filtered signal. The flow chart of the procedure is given in Fig. 12. Figure 13 shows the results of the optimization process for one case study.

The difference between the present and the earlier [1] study is that the ICF-based HPF is designed from the bivariate cubic B-spline here, whereas in ref. [1], the filters were designed from other model polynomial functions. Further, the evaluation method implemented is different, as the ICF-based HPF reported here is compared to the PSO-based HPF. Moreover, we have compared the k-space of three HPFs (ICF-based, traditional HPF, and PSO-based) to ascertain that the filtered images are significantly different.

### Contribution

Since its inception, the ICF has been used to study human brain tumors which are imaged with MRI [38] and human brain vasculature which is also imaged with MRI [39], and to mathematically characterize the input and output functions of the ICF-based filters using three different model polynomial functions [1]. The appearance of the ICF image as HPF signal demands an explanation. To this end, the main contribution of this study is the mathematical characterization of the ICF as an alternative HPF.

Moreover, this study provides two essential results. The first one is the reasoning that yields the understanding of the ICF formula. It is observed that the ICF embodies two main components: the image intensity and the gradients. The ICF can thus behave according to the prevalence of one component over the other. The prevalence is obtained when: (1) the numerical value of the image intensity is larger than that of the gradients and in such a case the ICF appears as a departing image; or (2) vice-versa and in such case the ICF appears as HP filtered signal.

The second contribution is the comparison of the ICF-based filter with the traditional HPF and the PSO-based HPF. The comparison is carried out in both image space and k-space, and it indicates that the three filters behave differently. Although data provided in this paper does not allow generalization, traditional HPF filtering and ICF-based filtering are found to be superior to PSO-based filtering. Further, the images filtered with the traditional HPF are found to be sharper than that with the ICF-based filter.

## Conclusion

In order to provide a mathematical formulation for the characterization of the HPF, the theory section of this paper presents the steps undertaken and the benefits obtained from an earlier research [1]. The first step is to include the ICF into the expression of the TF of the filter as the output function [see ΔE(x, y) in eq. (12)]. The second step consists of including the image pixel intensity as the input function of the TF of the filter [see f(0, 0) in eq. (12)]. The solution of this equation in x[n] (filter input) and y[n] (filter output) is outlined in the mathematical procedure, which starts from eq. (13) and ends at eqs. (15) and (16) for x[n] and y[n], respectively. This mathematical formalism allows the ICF to be classified as a HPF. The validity of the formalism, and thus the practical evidence of the fact that the ICF is an alternative HPF, is shown in Figs. 7 and 8. In conclusion, this paper provides a robust mathematical procedure to design a 2D HPF from a model polynomial function.

## Data Availability

The datasets used and analyzed during the current study are available from the corresponding author on reasonable request.
